# Genetic Association of the *KLK4* Locus with Risk of Prostate Cancer

**DOI:** 10.1371/journal.pone.0044520

**Published:** 2012-09-06

**Authors:** Felicity Lose, Srilakshmi Srinivasan, Tracy O’Mara, Louise Marquart, Suzanne Chambers, Robert A. Gardiner, Joanne F. Aitken, the Australian Prostate Cancer BioResource, Trina Yeadon, Pamela Saunders, Allison Eckert, Judith Clements, Peter Heathcote, Glenn Wood, Greg Malone, Hema Samaratunga, Angus Collins, Megan Turner, Kris Kerr, Amanda B. Spurdle, Jyotsna Batra, Judith A. Clements

**Affiliations:** Institute of Health and Biomedical Innovation, Queensland University of Technology, Brisbane, Queensland, Australia; Institute of Health and Biomedical Innovation, Queensland University of Technology, Brisbane, Queensland, Australia; Institute of Health and Biomedical Innovation, Queensland University of Technology, Brisbane, Queensland, Australia; Institute of Health and Biomedical Innovation, Queensland University of Technology, Brisbane, Queensland, Australia; Brisbane Urology Clinic, Brisbane Urology Clinic Central Queensland Urology Clinic, Wickham Terrace, Brisbane, Queensland, Australia; Brisbane Urology Clinic, Brisbane Urology Clinic Central Queensland Urology Clinic, Wickham Terrace, Brisbane, Queensland, Australia; Brisbane Urology Clinic, Brisbane Urology Clinic Central Queensland Urology Clinic, Wickham Terrace, Brisbane, Queensland, Australia; Aquesta Pathology, Toowong, Queensland, Australia; Sullivan and Nicolaides Pathology, Brisbane, Queensland, Australia; Sullivan and Nicolaides Pathology, Brisbane, Queensland, Australia; Sullivan and Nicolaides Pathology, Brisbane, Queensland, Australia; 1 Molecular Cancer Epidemiology Group, Genetics and Population Health Division, Queensland Institute of Medical Research, 300 Herston Rd, Herston, Brisbane, Queensland, Australia; 2 Australian Prostate Cancer Research Centre – Queensland, Institute of Health and Biomedical Innovation, Queensland University of Technology, Brisbane, Queensland, Australia; 3 Statistics Unit, Queensland Institute of Medical Research, Brisbane, Queensland, Australia; 4 Griffith Health Institute, Griffith University, Brisbane, Queensland, Australia; 5 Viertel Centre for Cancer Research, Cancer Council Queensland, Brisbane, Queensland, Australia; 6 University of Queensland Centre for Clinical Research, Royal Brisbane Hospital, Brisbane, Queensland, Australia; 7 Institute of Health and Biomedical Innovation, Queensland University of Technology, Brisbane, Queensland, Australia; University of Colorado, United States of America

## Abstract

The Kallikrein-related peptidase, KLK4, has been shown to be significantly overexpressed in prostate tumours in numerous studies and is suggested to be a potential biomarker for prostate cancer. KLK4 may also play a role in prostate cancer progression through its involvement in epithelial-mesenchymal transition, a more aggressive phenotype, and metastases to bone. It is well known that genetic variation has the potential to affect gene expression and/or various protein characteristics and hence we sought to investigate the possible role of single nucleotide polymorphisms (SNPs) in the *KLK4* gene in prostate cancer. Assessment of 61 SNPs in the *KLK4* locus (±10 kb) in approximately 1300 prostate cancer cases and 1300 male controls for associations with prostate cancer risk and/or prostate tumour aggressiveness (Gleason score <7 versus ≥7) revealed 7 SNPs to be associated with a decreased risk of prostate cancer at the P_trend_<0.05 significance level. Three of these SNPs, rs268923, rs56112930 and the HapMap tagSNP rs7248321, are located several kb upstream of *KLK4*; rs1654551 encodes a non-synonymous serine to alanine substitution at position 22 of the long isoform of the KLK4 protein, and the remaining 3 risk-associated SNPs, rs1701927, rs1090649 and rs806019, are located downstream of *KLK4* and are in high linkage disequilibrium with each other (r^2^≥0.98). Our findings provide suggestive evidence of a role for genetic variation in the *KLK4* locus in prostate cancer predisposition.

## Introduction

The *Kallikrein* (*KLK*) gene family consists of 15 genes in a tightly clustered locus over 320 kilobases (kb) at 19 q13.4 [1]. Many of the KLKs display altered expression in disease, in particular hormone-dependent cancers [1], [2]. KLK4 is hormone-regulated and is expressed predominantly in the prostate [3], [4], and to a lesser extent in other tissues [4], [5]. KLK4 has gained support as a potential biomarker for several hormone-dependent cancers [2], and for prostate cancer specifically, in that numerous studies have found KLK4 to be significantly overexpressed in prostate carcinoma tissues compared to benign prostatic hyperplasia [6] and normal tissues [7]–[11]. Of note, KLK4 is known to be expressed as a variety of isoforms [12], with the full length protein (254 amino acids long) showing the potential to be a better biomarker of prostate tumour cells than the commonly expressed shorter isoform (205 amino acids) [11]. In addition, KLK4 has been proposed to play a role in prostate cancer progression through its involvement in epithelial-mesenchymal transition [13], a more aggressive phenotype, and metastases to bone [14]. KLK4 overexpression has been reported to be associated with prostate cancer stage, although the direction of effect differed for *KLK4* mRNA (associated with advanced stage) [6] versus KLK4 protein (early stage tumours) [15].

Approximately 40% of prostate cancer is estimated to have a genetic component (http://www.genome.gov/gwastudies/) [16], and to date single nucleotide polymorphisms (SNPs) in over 40 loci have been identified by genome-wide association studies (GWAS) to be associated with prostate cancer risk [17]. One of these SNPs is located in the *KLK* locus, downstream of the *KLK3* gene [18], [19], [20], and is thought to be a marker for a potentially functional non-synonymous SNP within the *KLK3* gene [21]. Although no SNPs in *KLK4* have been reported by GWAS to be associated with prostate cancer at genome-wide significance levels to date, commonly used GWAS chips only capture 22% [22] - 44% [23] of validated genetic variation in the locus with r^2^≥0.80. Hence we sought to comprehensively investigate the role of *KLK4* in prostate cancer risk and tumour aggressiveness by genotyping the majority of validated genetic variation (±10 kb) around the *KLK4* locus in a large prostate cancer study group and male controls not screened for PSA levels.

## Materials and Methods

### Study Subjects

Study subjects have been described elsewhere [24], [25]. Briefly, from 2004 onwards, 1349 histopathologically-confirmed prostate cancer cases were recruited through private and public urologists in Queensland, Australia via three prostate cancer studies or resources: the Retrospective Queensland Study (N = 154; [26]), the Prostate Cancer Supportive Care and Patient Outcomes Project (ProsCan, N = 857; [25]) and from the Australian Prostate Cancer BioResource (APCB, N = 338; http://www.apccbioresource.org.au/index.html). Men presented to urologists with lower urinary tract symptoms and/or abnormal serum Prostate Specific Antigen (PSA), and 72% of cases possessed prostate tumours of Gleason score 7 or above. Cases ranged in age at diagnosis from 40–88 years (median 63 years). Male controls (N = 1405) with no self-reported personal history of prostate cancer were randomly selected from the Australian Electoral Roll and age-matched (in 5 year groups) and post-code matched to cases (N = 569), or recruited through the Australian Red Cross Blood Services in Brisbane (N = 836). Controls were not screened for PSA levels and analyses excluded 50 controls with age at interview <40 years (the age of the youngest case); included controls ranged in age at interview from 40–89 years of age (median 62 years). All participants had self-reported European ethnicity and gave written informed consent. The study protocol was approved by the Human Research Ethics Committees of the Queensland University of Technology, Queensland Institute of Medical Research, the Mater Hospital (for Brisbane Private Hospital), the Royal Brisbane Hospital, Princess Alexandra Hospital and the Cancer Council Queensland.

### SNP Selection and Genotyping

The *KLK4* gene region used for SNP selection was chr19∶56091420…56115806 (hg18), which encompasses the longest *KLK4* isoform ±10 kb. All SNPs in this region were extracted from the National Center for Biotechnology Information (NCBI) dbSNP build 130 [27], CHIP SNPper [28] and the “ParSNPs” database [29] and duplicates removed. SNPs not classified as validated were removed and validated SNPs were further investigated for occurrence in Europeans using SPSmart [30] and 1000 Genomes [23]. Additional SNPs excluded from investigation included all SNPs on the Illumina 550 K, 610 K and Omni1 genome-wide genotyping chips and SNPs assessed in the Cancer Genetics Markers of Susceptibility (CGEMS) project [31], unless there was evidence of association with prostate cancer by CGEMS (P<0.05). SNPs in high linkage disequilibrium (LD; r^2^≥0.80) with these excluded Illumina and CGEMS SNPs were also removed, determined by the SNP Annotation and Proxy Search program (SNAP) version 2.1 [32] using HapMap release 22 (1000 Genomes data was not available at the time of initiation of this study). We then prioritised for genotyping all independent SNPs (r^2^<0.80) according to SNAP using HapMap release 22 data (N = 74). An additional 8 *KLK4* tagSNPs (selected using HapMap data release 24/phase II, Nov 2008, NCBI build 36, dbSNP b126, using the Tagger program within Haploview v4.1 [33]), genotyped as part of a previous study, were also included (N = 82 overall).

SNPs were genotyped using iPLEX Gold assays on the Sequenom MassARRAY platform (Sequenom, San Diego, CA), as described previously [34]. There were 4 negative (H_2_O) controls per 384-well plate, and quality control parameters included genotype call rates >95%, a combination of cases and controls on each plate, inclusion of 20 duplicate samples per 384-well plate (>5% of samples) with ≥98% concordance between duplicates and Hardy-Weinberg Equilibrium *P* values >0.05. Of a total of 82 *KLK4* SNPs selected for investigation, 11 could not be designed for Sequenom assays, and after application of quality control parameters, 61 SNPs were successfully genotyped. After the study was completed, 1000 Genomes data became available and revealed that 6 *KLK4* SNPs not genotyped directly in our study (rs2659108, rs1654556, rs1090648, rs11881373, rs2569531 and rs73598979) were actually tagged by our genotyped SNPs (r^2^>0.80).

### Statistical Methods

Predictive Analytics Software (PASW) Statistics version 17.0.2 (SPSS Inc., Chicago, IL) was used for all analyses. Genotype and allele frequencies were calculated for the patient and control groups. SNP allele and genotype distributions were compared using χ^2^ and their association with prostate cancer susceptibility and clinical data were performed under codominant and linear models using logistic regression analysis. Prostate cancer cases with tumour Gleason scores ≥7 were classified as aggressive. All analyses were adjusted for age (as a continuous variable).

### SNP Function Prediction

Alibaba (http://labmom.com/link/alibaba_2_1_tf_binding_prediction), TFsearch (http://www.cbrc.jp/research/db/TFSEARCH.html) and MatInspector (http://www.genomatix.de/online_help/help_matinspector/matinspector_help.html) were used to predict the transcription factor binding sites. The program SignalP was used to predict the signal peptide (http://www.cbs.dtu.dk/services/SignalP/). miRNADA (http://www.microrna.org/microrna/home.do), Patrocles and (http://www.patrocles.org/) miRBase (http://www.mirbase.org/) were used to determine the effect of the SNP alleles on miRNA binding. JASPAR (http://jaspar.binf.ku.dk/), CISTER (http://zlab.bu.edu/~mfrith/cister.shtml) and NHRScan were used for the prediction of nuclear hormone receptor response elements. Splicing effects (using splice-finder), protein structure and stability (Polyphen, SIFT, SNP3D) were determined through the SNPinfo web server (http://snpinfo.niehs.nih.gov). Histone marks, DNAse hypersensitive sites and conservation scores were obtained from HaploReg (http://www.broadinstitute.org/mammals/haploreg/haploreg.php), which extracts data from the UCSC Browser (http://genome.ucsc.edu/). The F-SNP web server (http://compbio.cs.queensu.ca/F-SNP) was used to determine the functional score and putative effect of each SNP.

## Results

Seven SNPs were found to be monomorphic in our sample group (Table S1). Results of analyses of the remaining 54 *KLK4* SNPs and risk of prostate cancer are displayed in Table 1. Although no *KLK4* SNPs were statistically significantly associated with prostate cancer risk after Bonferroni correction (P<9×10^−4^), 7 SNPs were associated at the P_trend_<0.05 significance level and the majority of these displayed a modest decrease in prostate cancer risk of around 20%. Two of these SNPs, rs268923 (Odds Ratio (OR) 0.89, 95% Confidence Interval (CI) 0.79–1.00, P_trend_ = 0.045) and rs56112930 (OR 0.37, 95% CI 0.14–0.96, P_trend_ = 0.040; Minor Allele Frequency (MAF) 0.006), are located several kilobases upstream of the long isoform of *KLK4*, 8.2 kb and 6.5 kb, respectively. The *KLK4* tagSNP rs7248321 (OR 0.77, 95% CI 0.60–0.98, P_trend_ = 0.033; MAF 0.060), also represented on several of the genome-wide chips including the Illumina 550 K, 610 K and Omni1 chips, is located ∼4.5 kb upstream of *KLK4*, and rs7248321 tags two nearby SNPs rs13345980 and rs7246794 with r^2^ 1.00. rs1654551 (OR 0.79, 95% CI 0.65–0.97, P_trend_ = 0.023; MAF 0.093) is a non-synonymous SNP in the full length KLK4 protein coding for a serine to alanine amino acid (aa) substitution at position 22. In the more commonly expressed 205 aa KLK4 isoform, this SNP is located in the 5′ untranslated region [11]. The remaining three risk-associated SNPs, rs1701927, rs1090649 and rs806019, we determined to be in high LD with each other (r^2^≥0.98) and accordingly all display ORs of around 0.85 (95% CI range 0.73–1.00, P_trend_ range 0.030–0.044; MAF 0.175). Results were similar for rs1701926 that is also part of this high LD block (OR 0.86, 95% CI 0.74–1.00, P_trend_ = 0.058). All four SNPs are located downstream of the *KLK4* gene from 750 base pairs (bp) to 3.6 kb past the 3′ untranslated region (UTR).

**Table 1:** Association of *KLK4* SNPs and prostate cancer risk.

SNP	Genotype	Controls	Cases	Adjusted	
Chr position^a^		*n* (%)	*n* (%)	OR (95% CI)	P
rs17714461	GG	1000 (78.9)	984 (77.5)	1.00	
56116918	GA	255 (20.1)	277 (21.8)	1.11 (0.91–1.34)	0.307
	AA	12 (0.9)	9 (0.7)	0.77 (0.32–1.84)	0.556
	Per A allele			1.07 (0.89–1.28)	0.477
rs17714450	GG	1286 (99.3)	1250 (98.7)	1.00	
56115669	GA	9 (0.7)	17 (1.3)	1.94 (0.86–4.37)	0.112
	AA	0 (0.0)	0 (0.0)	*N/A*	*N/A*
	Per A allele			1.94 (0.86–4.37)	0.112
rs8101572	AA	395 (30.5)	389 (30.7)	1.00	
56115440	AC	619 (47.8)	634 (50.0)	1.03 (0.86–1.24)	0.726
	CC	281 (21.7)	245 (19.3)	0.87 (0.70–1.09)	0.236
	Per C allele			0.94 (0.85–1.05)	0.306
rs8100631	GG	489 (37.8)	474 (37.4)	1.00	
56115358	GA	586 (45.3)	592 (46.7)	1.03 (0.87–1.22)	0.731
	AA	220 (17.0)	201 (15.9)	0.93 (0.74–1.17)	0.535
	Per A allele			0.98 (0.87–1.09)	0.678
rs268920^c^	CC	1295 (100.0)	1267 (99.9)	1.00	
56114746	CG	0 (0.0)	1 (0.1)	*N/A*	*N/A*
	GG	0 (0.0)	0 (0.0)	*N/A*	*N/A*
	Per G allele			*N/A*	*N/A*
rs10427094	CC	1130 (88.9)	1113 (90.7)	1.00	
56114689	CT	137 (10.8)	112 (9.1)	0.84 (0.64–1.09)	0.183
	TT	4 (0.3)	2 (0.2)	0.53 (0.10–2.92)	0.466
	Per T allele			0.83 (0.64–1.06)	0.138
rs268921	CC	477 (36.9)	467 (36.8)	1.00	
56114503	CG	592 (45.7)	594 (46.8)	1.01 (0.85–1.20)	0.891
	GG	225 (17.4)	207 (16.3)	0.93 (0.74–1.17)	0.518
	Per G allele			0.97 (0.87–1.09)	0.613
**rs268923**	AA	416 (33.6)	450 (36.9)	1.00	
56114028	AT	617 (49.9)	593 (48.6)	0.88 (0.74–1.05)	0.159
	TT	204 (16.5)	176 (14.4)	0.79 (0.62–1.01)	0.059
	**Per T allele**			**0.89 (0.79–1.00)**	**0.045**
rs10419776	CC	940 (77.9)	692 (75.1)	1.00	
56113695	CG	249 (20.6)	216 (23.5)	1.17 (0.95–1.44)	0.137
	GG	18 (1.5)	13 (1.4)	0.90 (0.44–1.86)	0.780
	Per G allele			1.11 (0.93–1.34)	0.251
**rs56112930**	CC	1250 (98.7)	1234 (99.5)	1.00	
56112295	CT	16 (1.3)	6 (0.5)	0.37 (0.14–0.96)	0.040
	TT	0 (0.0)	0 (0.0)	*N/A*	*N/A*
	**Per T allele**			**0.37 (0.14–0.96)**	**0.040**
**rs7248321** b	AA	1182 (88.1)	1185 (90.7)	1.00	
56110317	AG	156 (11.6)	121 (9.3)	0.78 (0.60–1.00)	0.048
	GG	3 (0.2)	1 (0.1)	0.35 (0.04–3.35)	0.359
	**Per G allele**			**0.77 (0.60–0.98)**	**0.033**
rs2569526	AA	1259 (99.0)	1225 (99.7)	1.00	
56106299	AG	13 (1.0)	4 (0.3)	0.34 (0.11–1.06)	0.062
	GG	0 (0.0)	0 (0.0)	*N/A*	*N/A*
	Per G allele			0.34 (0.11–1.06)	0.062
rs2978642	TT	747 (57.7)	731 (57.8)	1.00	
56105718	TA	466 (36.0)	452 (35.7)	0.99 (0.84–1.17)	0.936
	AA	81 (6.3)	82 (6.5)	1.03 (0.74–1.42)	0.858
	Per A allele			1.00 (0.88–1.14)	0.947
rs198969^b^	GG	366 (27.3)	349 (26.7)	1.00	
56105614	GC	640 (47.8)	642 (49.1)	1.06 (0.88–1.27)	0.557
	CC	334 (24.9)	316 (24.2)	0.98 (0.79–1.22)	0.876
	Per C allele			0.99 (0.89–1.10)	0.897
rs2242669	CC	860 (68.0)	846 (68.3)	1.00	
56105602	CT	364 (28.8)	358 (28.9)	1.00 (0.84–1.19)	0.995
	TT	41 (3.2)	35 (2.8)	0.85 (0.54–1.35)	0.500
	Per T allele			0.97 (0.84–1.13)	0.708
rs198968^b^	CC	830 (62.8)	838 (64.4)	1.00	
56105140	CT	432 (32.7)	421 (32.4)	0.96 (0.81–1.13)	0.637
	TT	59 (4.5)	42 (3.2)	0.71 (0.47–1.07)	0.100
	Per T allele			0.91 (0.80–1.05)	0.195
rs198967	CC	1292 (99.9)	1265 (99.8)		
56104833	CT	1 (0.1)	3 (0.2)	3.73 (0.39–36.07)	0.255
	TT	0 (0.0)	0 (0.0)	*N/A*	*N/A*
	Per T allele			3.73 (0.39–36.07)	0.255
**rs1654551**	TT	1060 (81.9)	1082 (85.3)	1.00	
56104480	TG	227 (17.5)	181 (14.5)	0.79 (0.64–0.97)	0.028
	GG	7 (0.5)	5 (0.4)	0.67 (0.21–2.13)	0.499
	**Per G allele**			**0.79 (0.65–0.97)**	**0.023**
rs1654552	GG	403 (31.1)	376 (29.7)	1.00	
56104478	GT	603 (46.6)	637 (50.3)	1.15 (0.96–1.37)	0.140
	TT	288 (22.3)	254 (20.0)	0.95 (0.76–1.19)	0.658
	Per T allele			0.99 (0.89–1.10)	0.847
rs2242670^b^	CC	418 (31.2)	359 (27.4)	1.00	
56104127	CT	634 (47.4)	674 (51.5)	1.24 (1.04–1.48)	0.017
	TT	286 (21.4)	277 (21.1)	1.12 (0.90–1.39)	0.309
	Per T allele			1.07 (0.96–1.19)	0.210
rs198966	CC	1294 (99.9)	1265 (99.8)	1.00	
56103822	CT	1 (0.1)	2 (0.2)	2.47 (0.22–27.42)	0.460
	TT	0 (0.0)	1 (0.1)	*N/A*	*N/A*
	Per T allele			3.39 (0.45–25.58)	0.236
rs34626614	GG	1294 (99.9)	1267 (99.9)	1.00	
56103563	GA	1 (0.1)	1 (0.1)	1.18 (0.07–18.86)	0.909
	AA	0 (0.0)	0 (0.0)	*N/A*	*N/A*
	Per A allele			1.18 (0.07–18.86)	0.909
rs2569527	AA	1274 (98.4)	1255 (99.0)	1.00	
56103448	AC	21 (1.6)	13 (1.0)	0.62 (0.31–1.25)	0.186
	CC	0 (0.0)	0 (0.0)	*N/A*	*N/A*
	Per C allele			0.62 (0.31–1.25)	0.186
rs189903^c^	CC	1258 (100.0)	1184 (99.8)	1.00	
56103377	CT	0 (0.0)	2 (0.2)	*N/A*	*N/A*
	TT	0 (0.0)	0 (0.0)	*N/A*	*N/A*
	Per T allele			*N/A*	*N/A*
rs2979451^b^	AA	686 (52.0)	625 (48.2)	1.00	
56103200	AG	507 (38.4)	562 (43.3)	1.21 (1.03–1.42)	0.021
	GG	127 (9.6)	111 (8.6)	0.96 (0.73–1.27)	0.772
	Per G allele			1.06 (0.95–1.20)	0.298
rs1701929^b^	TT	703 (52.8)	721 (55.0)	1.00	
56103141	TC	540 (40.6)	520 (39.6)	0.94 (0.80–1.10)	0.411
	CC	88 (6.6)	71 (5.4)	0.77 (0.56–1.08)	0.130
	Per C allele			0.91 (0.80–1.03)	0.135
rs7255024	CC	1070 (88.2)	1083 (88.3)	1.00	
56103075	CA	140 (11.5)	140 (11.4)	0.99 (0.77–1.27)	0.912
	AA	3 (0.2)	4 (0.3)	1.36 (0.30–6.12)	0.687
	Per A allele			1.00 (0.79–1.27)	0.986
rs1654553^b^	AA	390 (29.2)	344 (26.3)	1.00	
56102928	AG	626 (46.9)	679 (51.9)	1.24 (1.04–1.49)	0.019
	GG	320 (24.0)	286 (21.8)	1.03 (0.83–1.27)	0.821
	Per G allele			1.02 (0.92–1.14)	0.687
rs2235091^b^	TT	583 (43.6)	520 (39.6)	1.00	
56102283	TC	563 (42.1)	609 (46.4)	1.21 (1.02–1.42)	0.025
	CC	192 (14.3)	183 (13.9)	1.06 (0.84–1.35)	0.606
	Per C allele			1.07 (0.96–1.20)	0.208
rs35945487	CC	1262 (97.5)	1238 (97.6)	1.00	
56102098	CT	32 (2.5)	30 (2.4)	0.93 (0.56–1.55)	0.790
	TT	0 (0.0)	0 (0.0)	*N/A*	*N/A*
	Per T allele			0.93 (0.56–1.55)	0.790
rs73042387	GG	884 (73.7)	901 (74.6)	1.00	
56101983	GA	291 (24.3)	287 (23.8)	0.96 (0.80–1.16)	0.686
	AA	25 (2.1)	20 (1.7)	0.80 (0.44–1.45)	0.460
	Per A allele			0.94 (0.80–1.11)	0.484
rs1139132	CC	540 (44.9)	488 (40.2)	1.00	
56101575	CA	499 (41.4)	563 (46.4)	1.24 (1.04–1.47)	0.015
	AA	165 (13.7)	163 (13.4)	1.08 (0.84–1.38)	0.555
	Per A allele			1.09 (0.97–1.22)	0.152
**rs1701927**	AA	882 (68.1)	906 (71.5)	1.00	
56100654	AC	374 (28.9)	336 (26.5)	0.87 (0.73–1.03)	0.110
	CC	39 (3.0)	26 (2.1)	0.65 (0.39–1.07)	0.091
	**Per C allele**			**0.85 (0.73–0.98)**	**0.030**
rs1701926	TT	863 (68.3)	871 (71.1)	1.00	
56100570	TG	363 (28.7)	332 (27.1)	0.91 (0.76–1.08)	0.273
	GG	37 (2.9)	22 (1.8)	0.58 (0.34–1.00)	0.050
	Per G allele			0.86 (0.74–1.00)	0.058
rs2569530	GG	334 (30.1)	274 (25.6)	1.00	
56100420	GT	509 (45.9)	553 (51.6)	1.33 (1.09–1.62)	0.006
	TT	265 (23.9)	244 (22.8)	1.12 (0.89–1.42)	0.332
	Per T allele			1.07 (0.95–1.20)	0.258
**rs1090649**	CC	860 (68.3)	871 (71.0)	1.00	
56098343	CG	358 (28.4)	333 (27.2)	0.92 (0.77–1.10)	0.344
	GG	41 (3.3)	22 (1.8)	0.53 (0.31–0.89)	0.017
	**Per G allele**			**0.86 (0.73–1.00)**	**0.044**
rs11881354	AA	557 (44.1)	504 (40.6)	1.00	
56097941	AG	531 (42.0)	574 (46.3)	1.19 (1.00–1.41)	0.045
	GG	176 (13.9)	162 (13.1)	1.01 (0.79–1.29)	0.945
	Per G allele			1.05 (0.94–1.18)	0.394
**rs806019**	CC	880 (68.0)	903 (71.2)	1.00	
56097794	CG	375 (29.0)	339 (26.7)	0.87 (0.73–1.04)	0.129
	GG	39 (3.0)	26 (2.1)	0.65 (0.39–1.07)	0.092
	**Per G allele**			**0.85 (0.73–0.99)**	**0.036**
rs806020	GG	365 (28.9)	326 (26.4)	1.00	
56097620	GA	591 (46.9)	632 (51.2)	1.21 (1.00–1.46)	0.049
	AA	305 (24.2)	277 (22.4)	1.03 (0.82–1.28)	0.802
	Per A allele			1.02 (0.91–1.14)	0.704
rs806021	AA	374 (29.6)	338 (27.3)	1.00	
56097485	AG	592 (46.9)	625 (50.5)	1.17 (0.97–1.41)	0.093
	GG	297 (23.5)	274 (22.2)	1.03 (0.82–1.28)	0.802
	Per G allele			1.02 (0.92–1.14)	0.696
rs806022	GG	378 (29.2)	332 (26.2)	1.00	
56096999	GT	610 (47.1)	650 (51.3)	1.22 (1.01–1.46)	0.037
	TT	306 (23.6)	285 (22.5)	1.07 (0.86–1.33)	0.559
	Per T allele			1.04 (0.93–1.16)	0.473
rs806023	AA	379 (29.4)	342 (27.1)	1.00	
56096896	AG	608 (47.1)	640 (50.7)	1.17 (0.97–1.40)	0.097
	GG	303 (23.5)	281 (22.2)	1.03 (0.83–1.29)	0.766
	Per G allele			1.02 (0.92–1.14)	0.669
rs2569535	GG	1278 (98.9)	1256 (99.5)	1.00	
56096639	GC	14 (1.1)	6 (0.5)	0.48 (0.18–1.24)	0.130
	CC	0 (0.0)	0 (0.0)	*N/A*	*N/A*
	Per C allele			0.48 (0.18–1.24)	0.130
rs1701941	TT	357 (27.8)	316 (25.0)	1.00	
56096032	TA	606 (47.9)	650 (51.4)	1.19 (0.99–1.44)	0.065
	AA	312 (24.3)	298 (23.6)	1.08 (0.87–1.35)	0.478
	Per A allele			1.04 (0.94–1.17)	0.441
rs1654513	AA	550 (42.5)	555 (43.8)	1.00	
56094494	AG	593 (45.8)	578 (45.6)	0.97 (0.82–1.14)	0.701
	GG	152 (11.7)	135 (10.6)	0.88 (0.68–1.14)	0.341
	Per G allele			0.95 (0.84–1.07)	0.374
rs8104538	TT	621 (48.0)	606 (47.8)	1.00	
56093609	TC	542 (41.9)	535 (42.2)	1.00 (0.85–1.18)	0.978
	CC	132 (10.2)	126 (9.9)	0.98 (0.75–1.28)	0.883
	Per C allele			0.99 (0.88–1.12)	0.926
rs10409668^c^	CC	1295 (100.0)	1267 (99.9)	1.00	
56093213	CT	0 (0.0)	1 (0.1)	*N/A*	*N/A*
	TT	0 (0.0)	0 (0.0)	*N/A*	*N/A*
	Per T allele			*N/A*	*N/A*
rs1560719	TT	521 (40.4)	475 (38.3)	1.00	
56092648	TC	569 (44.1)	581 (46.8)	1.12 (0.95–1.33)	0.178
	CC	201 (15.6)	185 (14.9)	1.01 (0.80–1.27)	0.956
	Per C allele			1.03 (0.92–1.15)	0.614
rs2739493	TT	489 (37.8)	435 (34.4)	1.00	
56092488	TG	583 (45.1)	609 (48.1)	1.17 (0.98–1.39)	0.076
	GG	221 (17.1)	221 (17.5)	1.11 (0.88–1.39)	0.368
	Per G allele			1.07 (0.96–1.20)	0.210
rs1701934	GG	1283 (99.1)	1256 (99.1)	1.00	
56092213	GA	12 (0.9)	11 (0.9)	0.97 (0.43–2.21)	0.944
	AA	0 (0.0)	1 (0.1)	*N/A*	*N/A*
	Per A allele			1.15 (0.54–2.46)	0.716
rs1560723	TT	1282 (99.1)	1255 (99.1)	1.00	
56092168	TC	12 (0.9)	11 (0.9)	0.97 (0.43–2.21)	0.944
	CC	0 (0.0)	1 (0.1)	*N/A*	*N/A*
	Per C allele			1.15 (0.54–2.46)	0.716
rs1701936	CC	1292 (99.8)	1263 (99.7)	1.00	
56091945	CT	3 (0.2)	3 (0.2)	1.17 (0.23–5.83)	0.849
	TT	0 (0.0)	1 (0.1)	*N/A*	*N/A*
	Per T allele			1.74 (0.47–6.42)	0.405
rs1654514	AA	1282 (99.0)	1256 (99.1)	1.00	
56091856	AG	13 (1.0)	11 (0.9)	0.89 (0.40–2.00)	0.780
	GG	0 (0.0)	1 (0.1)	*N/A*	*N/A*
	Per G allele			1.06 (0.50–2.24)	0.871
rs1701937	CC	1292 (99.8)	1264 (99.7)	1.00	
56091743	CA	3 (0.2)	3 (0.2)	1.17 (0.23–5.82)	0.850
	AA	0 (0.0)	1 (0.1)	*N/A*	*N/A*
	Per A allele			1.74 (0.47–6.41)	0.406

SNP, single nucleotide polymorphism; chr, chromosome; n, number; OR, odds ratio; CI, confidence interval.

a Co-ordinates from hg18.

b tagSNP.

c SNP too infrequent in these groups to calculate OR (95% CI).

**Bold**, SNPs displaying a P_trend_ value <0.05.

Only one *KLK4* SNP, rs198968, was associated with prostate tumour aggressiveness (Gleason score <7 *vs*. ≥7: OR 0.76 (95% CI 0.60–0.95, P_trend_ = 0.016; Table 2). However, this result was not reflected in a more robust Gleason score analysis comparing “extreme” Gleason categories, ≤6 (N = 329) *vs.* ≥8 (N = 173), with an odds ratio of 0.95 (95% CI 0.68–1.32; P_trend_ = 0.752).

**Table 2:** Association of *KLK4* SNPs and prostate tumour aggressiveness.

SNP	Genotype	Gleason <7	Gleason ≥7	Adjusted	
Chr position^a^		*n* (%)	*n* (%)	OR (95% CI)	P
rs17714461	GG	241 (75.1)	640 (76.9)	1.00	
56116918	GA	77 (24.0)	257 (22.4)	0.86 (0.63–1.17)	0.340
	AA	3 (0.9)	7 (0.6)	0.60 (0.13–2.73)	0.510
	Per A allele			0.85 (0.64–1.13)	0.269
rs17714450	GG	316 (98.8)	813 (98.7)	1.00	
56115669	GA	4 (1.3)	11 (1.3)	1.05 (0.33–3.34)	0.936
	AA	0 (0.0)	0 (0.0)	*N/A*	*N/A*
	Per A allele			1.05 (0.33–3.34)	0.936
rs8101572	AA	106 (33.1)	246 (29.8)	1.00	
56115440	AC	154 (48.1)	417 (50.5)	1.14 (0.85–1.53)	0.397
	CC	60 (18.8)	162 (19.6)	1.12 (0.77–1.63)	0.563
	Per C allele			1.07 (0.89–1.29)	0.494
rs8100631	GG	125 (39.1)	301 (36.5)	1.00	
56115358	GA	145 (45.3)	393 (47.7)	1.10 (0.83–1.46)	0.506
	AA	50 (15.6)	130 (15.8)	1.05 (0.71–1.55)	0.817
	Per A allele			1.04 (0.86–1.25)	0.685
rs268920^c^	CC	320 (100.0)	824 (99.9)	1.00	
56114746	CG	0 (0.0)	1 (0.1)	*N/A*	*N/A*
	GG	0 (0.0)	0 (0.0)	*N/A*	*N/A*
	Per G allele			*N/A*	*N/A*
rs10427094	CC	292 (91.8)	717 (90.4)	1.00	
56114689	CT	26 (8.2)	75 (9.5)	1.16 (0.72–1.85)	0.540
	TT	0 (0.0)	1 (0.1)	*N/A*	*N/A*
	Per T allele			1.19 (0.75–1.89)	0.466
rs268921	CC	123 (38.4)	297 (36.0)	1.00	
56114503	CG	144 (45.0)	395 (47.9)	1.11 (0.83–1.48)	0.476
	GG	53 (16.6)	133 (16.1)	1.01 (0.69–1.48)	0.974
	Per G allele			1.02 (0.85–1.23)	0.806
rs268923	AA	113 (36.8)	297 (37.4)	1.00	
56114028	AT	140 (45.6)	390 (49.1)	1.04 (0.78–1.40)	0.778
	TT	54 (17.6)	108 (13.6)	0.75 (0.51–1.12)	0.163
	Per T allele			0.90 (0.74–1.09)	0.293
rs10419776	CC	190 (79.2)	414 (73.8)	1.00	
56113695	CG	49 (20.4)	138 (24.6)	1.23 (0.85–1.79)	0.273
	GG	1 (0.4)	9 (1.6)	3.70 (0.46–29.58)	0.217
	Per G allele			1.31 (0.93–1.85)	0.128
rs56112930	CC	312 (99.7)	804 (99.6)	1.00	
56112295	CT	1 (0.3)	3 (0.4)	1.34 (0.14–13.06)	0.803
	TT	0 (0.0)	0 (0.0)	*N/A*	*N/A*
	Per T allele			1.34 (0.14–13.06)	0.803
rs7248321^b^	AA	303 (90.4)	762 (91.0)	1.00	
56110317	AG	31 (9.3)	75 (9.0)	0.93 (0.60–1.44)	0.741
	GG	1 (0.3)	0 (0.0)	*N/A*	*N/A*
	Per G allele			0.87 (0.56–1.33)	0.516
rs2569526	AA	318 (99.7)	792 (99.7)	1.00	
56106299	AG	1 (0.3)	2 (0.3)	1.14 (0.10–12.82)	0.913
	GG	0 (0.0)	0 (0.0)	*N/A*	*N/A*
	Per G allele			1.14 (0.1–12.82)	0.913
rs2978642	TT	184 (57.9)	475 (57.6)	1.00	
56105718	TA	112 (35.2)	297 (36.0)	1.02 (0.77–1.35)	0.893
	AA	22 (6.9)	52 (6.3)	0.94 (0.55–1.59)	0.806
	Per A allele			0.99 (0.80–1.22)	0.938
rs198969^b^	GG	84 (25.1)	231 (27.6)	1.00	
56105614	GC	166 (49.6)	404 (48.3)	0.87 (0.64–1.19)	0.382
	CC	85 (25.4)	202 (24.1)	0.84 (0.59–1.20)	0.333
	Per C allele			0.91 (0.77–1.09)	0.328
rs2242669	CC	209 (66.8)	557 (69.1)	1.00	
56105602	CT	96 (30.7)	224 (27.8)	0.87 (0.65–1.15)	0.326
	TT	8 (2.6)	26 (3.1)	1.16 (0.51–2.62)	0.728
	Per T allele			0.93 (0.73–1.19)	0.560
**rs198968** b	CC	199 (60.5)	553 (66.0)	1.00	
56105140	CT	111 (33.7)	266 (31.7)	0.87 (0.66–1.15)	0.342
	TT	19 (5.8)	19 (2.3)	0.36 (0.18–0.69)	0.002
	**Per T allele**			**0.76 (0.60–0.95)**	**0.016**
rs198967	CC	319 (99.7)	824 (99.9)	1.00	
56104833	CT	1 (0.3)	1 (0.1)	0.56 (0.03–9.16)	0.686
	TT	0 (0.0)	0 (0.0)	*N/A*	*N/A*
	Per T allele			0.56 (0.03–9.16)	0.686
rs1654551	TT	269 (84.1)	709 (85.9)	1.00	
56104480	TG	50 (15.6)	112 (13.6)	0.84 (0.59–1.22)	0.364
	GG	1 (0.3)	4 (0.5)	1.32 (0.14–12.00)	0.807
	Per G allele			0.87 (0.62–1.23)	0.439
rs1654552	GG	98 (30.6)	247 (30.0)	1.00	
56104478	GT	163 (50.9)	405 (49.2)	1.00 (0.74–1.35)	0.997
	TT	59 (18.4)	172 (20.9)	1.18 (0.81–1.73)	0.383
	Per T allele			1.08 (0.89–1.30)	0.431
rs2242670^b^	CC	90 (26.8)	236 (28.1)	1.00	
56104127	CT	172 (51.2)	425 (50.6)	0.95 (0.70–1.28)	0.722
	TT	74 (22.0)	179 (21.3)	0.91 (0.63–1.31)	0.607
	Per T allele			0.95 (0.79–1.14)	0.603
rs198966	CC	319 (99.7)	824 (99.9)	1.00	
56103822	CT	1 (0.3)	1 (0.1)	0.56 (0.03–9.16)	0.686
	TT	0 (0.0)	0 (0.0)	*N/A*	*N/A*
	Per T allele			0.56 (0.03–9.16)	0.686
rs34626614^c^	GG	320 (100.0)	825 (100.0)	1.00	
56103563	GA	0 (0.0)	0 (0.0)	*N/A*	*N/A*
	AA	0 (0.0)	0 (0.0)	*N/A*	*N/A*
	Per A allele			*N/A*	*N/A*
rs2569527	AA	317 (99.1)	817 (99.0)	1.00	
56103448	AC	3 (0.9)	8 (1.0)	0.96 (0.25–3.65)	0.948
	CC	0 (0.0)	0 (0.0)	*N/A*	*N/A*
	Per C allele			0.96 (0.25–3.65)	0.948
rs189903^c^	CC	299 (100.0)	762 (99.9)	1.00	
56103377	CT	0 (0.0)	1 (0.1)	*N/A*	*N/A*
	TT	0 (0.0)	0 (0.0)	*N/A*	*N/A*
	Per T allele			*N/A*	*N/A*
rs2979451^b^	AA	175 (53.2)	394 (47.1)	1.00	
56103200	AG	127 (38.6)	368 (44.0)	1.24 (0.94–1.63)	0.122
	GG	27 (8.2)	76 (9.0)	1.21 (0.75–1.95)	0.437
	Per G allele			1.16 (0.94–1.42)	0.159
rs1701929^b^	TT	180 (53.6)	460 (54.7)	1.00	
56103141	TC	132 (39.3)	341 (40.5)	1.02 (0.78–1.33)	0.882
	CC	24 (7.1)	40 (4.8)	0.62 (0.36–1.06)	0.080
	Per C allele			0.90 (0.73–1.11)	0.334
rs7255024	CC	272 (87.7)	696 (88.3)	1.00	
56103075	CA	36 (11.6)	91 (11.5)	1.00 (0.66–1.51)	0.996
	AA	2 (0.6)	1 (0.1)	0.19 (0.02–2.15)	0.181
	Per A allele			0.92 (0.62–1.35)	0.658
rs1654553*	AA	87 (26.0)	226 (26.9)	1.00	
56102928	AG	175 (52.2)	424 (50.5)	0.94 (0.69–1.28)	0.699
	GG	73 (21.8)	189 (22.5)	1.03 (0.71–1.49)	0.868
	Per G allele			1.01 (0.84–1.22)	0.895
rs2235091^b^	TT	146 (43.5)	329 (39.1)	1.00	
56102283	TC	145 (43.2)	387 (46.0)	1.15 (0.87–1.51)	0.325
	CC	45 (13.4)	125 (14.9)	1.21 (0.82–1.80)	0.338
	Per C allele			1.11 (0.93–1.34)	0.255
rs35945487	CC	312 (97.5)	806 (97.7)	1.00	
56102098	CT	8 (2.5)	19 (2.3)	0.96 (0.41–2.23)	0.921
	TT	0 (0.0)	0 (0.0)	*N/A*	*N/A*
	Per T allele			0.96 (0.41–2.23)	0.921
rs73042387	GG	228 (75.7)	584 (74.9)	1.00	
56101983	GA	64 (21.3)	187 (24.0)	1.13 (0.82–1.57)	0.452
	AA	9 (3.0)	9 (1.2)	0.40 (0.15–1.02)	0.055
	Per A allele			0.96 (0.72–1.26)	0.754
rs1139132	CC	126 (41.7)	326 (41.5)	1.00	
56101575	CA	137 (45.4)	351 (44.7)	0.99 (0.74–1.32)	0.931
	AA	39 (12.9)	109 (13.9)	1.05 (0.69–1.60)	0.818
	Per A allele			1.01 (0.83–1.23)	0.883
rs1701927	AA	223 (69.7)	592 (71.8)	1.00	
56100654	AC	88 (27.5)	218 (26.4)	0.93 (0.69–1.25)	0.622
	CC	9 (2.8)	15 (1.8)	0.60 (0.26–1.41)	0.241
	Per C allele			0.88 (0.68–1.13)	0.325
rs1701926	TT	217 (68.2)	568 (71.7)	1.00	
56100570	TG	93 (29.2)	211 (26.6)	0.87 (0.65–1.17)	0.359
	GG	8 (2.5)	13 (1.6)	0.60 (0.24–1.49)	0.272
	Per G allele			0.85 (0.65–1.09)	0.198
rs2569530	GG	67 (23.8)	181 (26.2)	1.00	
56100420	GT	148 (52.7)	352 (51.0)	0.87 (0.62–1.23)	0.438
	TT	66 (23.5)	157 (22.8)	0.89 (0.59–1.33)	0.570
	Per T allele			0.94 (0.77–1.15)	0.560
rs1090649	CC	217 (68.0)	567 (71.7)	1.00	
56098343	CG	94 (29.5)	211 (26.7)	0.87 (0.65–1.16)	0.330
	GG	8 (2.5)	13 (1.6)	0.60 (0.24–1.49)	0.273
	Per G allele			0.84 (0.65–1.09)	0.183
rs11881354	AA	135 (43.1)	328 (40.6)	1.00	
56097941	AG	139 (44.4)	368 (45.6)	1.05 (0.79–1.39)	0.726
	GG	39 (12.5)	111 (13.8)	1.15 (0.75–1.74)	0.521
	Per G allele			1.07 (0.88–1.29)	0.518
rs806019	CC	222 (69.4)	590 (71.5)	1.00	
56097794	CG	89 (27.8)	220 (26.7)	0.93 (0.69–1.24)	0.618
	GG	8 (2.8)	15 (1.8)	0.60 (0.26–1.41)	0.241
	Per G allele			0.88 (0.68–1.13)	0.323
rs806020	GG	82 (26.3)	216 (26.8)	1.00	
56097620	GA	164 (52.6)	402 (49.9)	0.94 (0.69–1.29)	0.692
	AA	66 (21.2)	187 (23.2)	1.11 (0.76–1.63)	0.579
	Per A allele			1.05 (0.87–1.27)	0.615
rs806021	AA	84 (26.8)	223 (27.7)	1.00	
56097485	AG	164 (52.4)	397 (49.3)	0.91 (0.67–1.25)	0.564
	GG	65 (20.8)	185 (23.0)	1.10 (0.75–1.61)	0.613
	Per G allele			1.04 (0.86–1.26)	0.669
rs806022	GG	83 (26.0)	220 (26.7)	1.00	
56096999	GT	165 (51.7)	417 (50.5)	0.95 (0.70–1.30)	0.763
	TT	71 (22.3)	188 (22.8)	1.03 (0.71–1.50)	0.884
	Per T allele			1.01 (0.84–1.22)	0.903
rs806023	AA	85 (26.7)	227 (27.6)	1.00	
56096896	AG	164 (51.6)	409 (49.8)	0.93 (0.68–1.27)	0.639
	GG	69 (21.7)	186 (22.6)	1.04 (0.71–1.51)	0.854
	Per G allele			1.01 (0.84–1.22)	0.891
rs2569535	GG	315 (99.1)	821 (99.9)	1.00	
56096639	GC	3 (0.9)	1 (0.1)	0.16 (0.02–1.56)	0.114
	CC	0 (0.0)	0 (0.0)	*N/A*	*N/A*
	Per C allele			0.16 (0.02–1.56)	0.114
rs1701941	TT	80 (25.1)	209 (25.4)	1.00	
56096032	TA	163 (51.1)	418 (50.9)	0.97 (0.71–1.34)	0.872
	AA	76 (23.8)	195 (23.7)	1.00 (0.69–1.45)	0.995
	Per A allele			1.00 (0.83–1.20)	0.998
rs1654513	AA	132 (41.3)	367 (44.5)	1.00	
56094494	AG	148 (46.3)	375 (45.5)	0.92 (0.70–1.21)	0.543
	GG	40 (12.5)	83 (10.1)	0.75 (0.49–1.15)	0.189
	Per G allele			0.88 (0.73–1.07)	0.208
rs8104538	TT	161 (50.3)	393 (47.7)	1.00	
56093609	TC	126 (39.4)	345 (41.9)	1.11 (0.84–1.47)	0.450
	CC	33 (10.3)	86 (10.4)	1.09 (0.70–1.70)	0.705
	Per C allele			1.07 (0.88–1.30)	0.512
rs10409668^c^	CC	320 (100.0)	824 (99.9)	1.00	
56093213	CT	0 (0.0)	1 (0.1)	*N/A*	*N/A*
	TT	0 (0.0)	0 (0.0)	*N/A*	*N/A*
	Per T allele			*N/A*	*N/A*
rs1560719	TT	129 (41.1)	301 (37.4)	1.00	
56092648	TC	137 (43.6)	383 (47.6)	1.18 (0.88–1.56)	0.269
	CC	48 (15.3)	121 (15.0)	1.07 (0.72–1.59)	0.746
	Per C allele			1.06 (0.88–1.29)	0.519
rs2739493	TT	112 (35.0)	276 (33.6)	1.00	
56092488	TG	153 (47.8)	401 (48.8)	1.03 (0.77–1.37)	0.857
	GG	55 (17.2)	145 (17.6)	1.04 (0.71–1.53)	0.832
	Per G allele			1.02 (0.85–1.23)	0.819
rs1701934	GG	316 (98.8)	819 (99.3)	1.00	
56092213	GA	4 (1.3)	6 (0.7)	0.66 (0.18–2.37)	0.521
	AA	0 (0.0)	0 (0.0)	*N/A*	*N/A*
	Per A allele			0.66 (0.18–2.37)	0.521
rs1560723	TT	316 (98.8)	818 (99.3)	1.00	
56092168	TC	4 (1.3)	6 (0.7)	0.66 (0.18–2.37)	0.522
	CC	0 (0.0)	0 (0.0)	*N/A*	*N/A*
	Per C allele			0.66 (0.18–2.37)	0.522
rs1701936	CC	318 (99.4)	823 (99.9)	1.00	
56091945	CT	2 (0.6)	1 (0.1)	0.28 (0.03–3.18)	0.307
	TT	0 (0.0)	0 (0.0)	*N/A*	*N/A*
	Per T allele			0.28 (0.03–3.18)	0.307
rs1654514	AA	316 (98.8)	819 (99.3)	1.00	
56091856	AG	4 (1.3)	6 (0.7)	0.66 (0.18–2.37)	0.521
	GG	0 (0.0)	0 (0.0)	*N/A*	*N/A*
	Per G allele			0.66 (0.18–2.37)	0.521
rs1701937	CC	318 (99.4)	824 (99.9)	1.00	
56091743	CA	2 (0.6)	1 (0.1)	0.28 (0.03–3.17)	0.306
	AA	0 (0.0)	0 (0.0)	*N/A*	*N/A*
	Per A allele			0.28 (0.03–3.17)	0.306

SNP, single nucleotide polymorphism; chr, chromosome; n, number; OR, odds ratio; CI, confidence interval.

a Co-ordinates from hg18.

b tagSNP;

c SNP too infrequent in these groups to calculate OR (95% CI).

**Bold**, SNPs displaying a P_trend_ value <0.05.

Results of bioinformatic prediction of functions of the associated SNPs are provided in Table S2. SNPs rs268923, rs198968, rs1654551, rs1701926, rs1090649 and rs806019 were found to alter transcription factor binding sites as predicted by at least one prediction tool. rs198968 and rs1654551 also lie within promoter histone marks as well as DNAse hypersensitive sites (Table S2), and hence are better candidate for functional follow up studies.

SNP rs1654551 leads to a serine to alanine amino acid change, but is predicted to be benign using the FASTSNP web server, although the SNP is predicted to effect o-glycosylation. In addition, PsortII prediction (http://urgi.versailles.inra.fr/Tools/PsortII) predicted the serine variant to be only 44.4% extracellularly localised as compared to the alanine variant which is predicted to be 55.6% extracellular. This is backed by SignalP predicting alteration of the KLK4 signal peptide sequence for the serine variant. Further, this SNP is also predicted to be involved in differential splicing.

## Discussion

We performed a comprehensive investigation of the role of variation in the *KLK4* gene in prostate cancer risk and/or tumour aggressiveness by assessing the majority of SNPs that have not been covered by previously performed GWA studies. Our study of approximately 1,300 cases and 1,300 male controls provided suggestive evidence that several *KLK4* SNPs may be associated with decreased risk of prostate cancer, and bioinformatic analysis provides evidence that some of these have potential biological relevance in prostate cancer.

None of the nominally risk-associated SNPs were located in known *KLK4* hormone response elements [35]. Three SNPs lay several kb upstream of the *KLK4* gene. rs7248321 is a tagSNP that has not been previously reported to be associated with prostate cancer risk in any GWAS, including CGEMS, and given the large numbers of samples assessed in previous studies [17], it is likely to be a false-positive result. Bioinformatic analyses of the rare rs56112930 SNP did not reveal any predicted effects on transcription factor binding sites [36], [37], [38]. SNP rs268923 was calculated by three different transcription factor binding site prediction programs to possibly have an effect [36]–[39], and although each program predicts different transcription factor binding sites to be altered by the SNP; one example of a prostate cancer relevant result is the predicted gain of an Oct-1 site [36]. Oct-1 is a known co-regulator of the androgen receptor [40], regulates growth of prostate cancer cells and is associated with poor prognosis [41].

The only SNP located in the *KLK4* coding region found to be marginally associated with prostate cancer risk was rs1654551. Since splicing of the *KLK4* locus is complex and results in several *KLK4* mRNA forms being produced [12], there are several possible functional consequences of this substitution. The two protein isoforms identified to date, in order of expression in normal prostate, are an intracellular 205 amino acid (aa) protein which lacks the classical KLK signal peptide and is localised to the nucleus (“short” isoform) [9], [11], and a secreted 254 aa protein that is cytoplasmically localised [11], [13]. rs1654551 codes for a serine to alanine substitution at amino acid 22 of the long isoform, or is located in the 5′ UTR of the 205 aa KLK4 protein. Although both the short and long isoforms have been found to be overexpressed in prostate cancer cells, the “long” 254 aa KLK4 protein is better able to discriminate between tumour and normal cells [11] and hence may be the more biologically relevant isoform in prostate cancer. Amino acid 22 is located within the signal peptide region of KLK4, which is cleaved off between aa 26 and 27 to result in secretion. It is unknown what the potential functional effects of an amino acid substitution are within the signal peptide. However, a recent study has shown that this cleaved peptide may be a useful target in prostate cancer immunotherapy, with the KLK4 signal peptide successfully inducing and expanding the cytotoxic T lymphocyte response more readily than PSA or Prostatic Acid Phosphotase (PAP) [42]. In addition, *in silico* analysis using the signal peptide prediction program SignalP [43] predicted a Serine22Alanine substitution to alter the cleavage site from aa 26/27 to aa 21/22. This would result in a KLK4 pro-protein with an additional 5 aa, which could potentially affect localisation or possibly even activation of the KLK4 proenzyme. Of relevance, a form of PSA has been reported that has an altered signal/pro-peptide and, although the pro-PSA sequence is truncated (not lengthened as is predicted for KLK4), the signal peptide alteration does result in an isoform of PSA that is unable to be activated [44]. This [-2]pro-PSA isoform is now also the basis of a commercially available prostate cancer serum test [45].

Attempting to predict the possible functional effects of the four associated SNPs located downstream of *KLK4*, rs1701927, rs1701926, rs1090649 and rs806019, is not as clearly directed. It is possible that some or all of these SNPs might alter enhancer/silencer binding sites, affecting expression of *KLK4*. *In silico* transcription factor binding site analysis predicts that rs1701926, rs1090649 and rs806019 may alter transcription factor binding sites [36]–[39] relevant to prostate cancer. The closest validated gene to the *KLK4* 3′ end is the Kallikrein family pseudogene *KLKP1*, thus these SNPs may regulate the activity of this pseudogene or expression of *KLKP1* transcripts, which have been shown to be down-regulated in prostate cancer tissues [46]. In addition, one other SNP not genotyped in this study, rs1654556, is in high LD (r^2^≥0.80) with these SNPs [23] and is predicted to alter mRNA folding [47] and miRNA binding (Table S2).

To the best of our knowledge, only two other studies have examined the role of *KLK4* SNPs in cancer (aside from genome-wide investigations). Recently Klein *et al*. investigated the effect of common variation in the exons and putative promoter regions of all 15 *KLK* genes on prostate cancer risk and levels of PSA forms and KLK2 [48]. Five *KLK4* SNPs – rs198969, rs198968, rs1654552, rs1654551 and rs1654553 - were assessed for association with prostate cancer risk in the Cancer Prostate in Sweden (CAPS) 1 sample set of over 1,400 cases and 700 controls and none were found to be associated. A second study in a small Korean sample set of 117 breast cancer cases and 194 controls found *KLK4* SNP rs806019 to be associated with a decreased risk of breast cancer (Odds Ratio 0.53; 95% Confidence Interval 0.33–0.85; P = 0.007) [49], a finding of similar magnitude and direction to that observed in our study of prostate cancer. Part of our study design was to exclude *KLK4* SNPs already assessed in GWAS, except for those reported to be associated with prostate cancer at the P<0.05 level in CGEMS. Four SNPs in CGEMS gave evidence of association with prostate cancer - rs17714461, rs8101572, rs8100631 and rs10427094 [31]. All four were genotyped in this study, but none were associated in our sample set. Of note, rs17714461 was recently reported to interact with the GWAS-detected *KLK2/3* SNP rs2735839 in CGEMS [50]. The authors mention that this result is notable considering KLK4 and KLK2 collaborate to stimulate cellular proliferation in prostate cancer [51]. rs2735839 genotype data was available for only a small proportion of our samples and hence we did not investigate this interaction further.

Our well-sized study indicates a possible contribution of SNPs in the *KLK4* gene to decreased risk of prostate cancer. However, these results should be interpreted cautiously considering the number of tests performed, and validation in much larger sample sets such as those of the PRACTICAL consortium is necessary.

## Supporting Information

Table S1 **rs IDs found to be monomorphic in this study.**
(DOC)

Table S2 ***In silico*** **function prediction of prostate cancer associated KLK4 gene variants.**
(XLSX)
