# Supplementary material for: Genetic Association of the KLK4 Locus with Risk of Prostate Cancer
Source: PLoS One. 2012 Sep 6;7(9):e44520. doi: 10.1371/journal.pone.0044520 (PMC3435290; doi:10.1371/journal.pone.0044520)
Supplement: Table S1 — rs IDs found to be monomorphic in this study. (DOC) [file pone.0044520.s001.doc]

**Table S1.** rs IDs found to be monomorphic in this study

| SNP | Reference allele | Controls (*n*) | Cases (*n*) |
| --- | --- | --- | --- |
| rs11670134 | G | 1295 | 1268 |
| rs10403424 | A | 1071 | 780 |
| rs3760736 | T | 1295 | 1268 |
| rs806024 | C | 1294 | 1266 |
| rs9304705 | C | 1295 | 1268 |
| rs11667106 | C | 1259 | 1187 |
| rs1610231 | C | 1294 | 1265 |

SNP, single nucleotide polymorphism; n, number.
